# Semantic intrusion errors are associated with plasma Ptau-181 among persons with amnestic mild cognitive impairment who are amyloid positive

**DOI:** 10.3389/fneur.2023.1179205

**Published:** 2023-08-04

**Authors:** Rosie E. Curiel Cid, Alexandra Ortega, Elizabeth A. Crocco, Diana Hincapie, Karen N. McFarland, Ranjan Duara, David Vaillancourt, Steven T. DeKosky, Glenn Smith, Efrosyni Sfakianaki, Monica Rosselli, Warren W. Barker, Malek Adjouadi, Yarlenis Barreto, Yuleidys Feito, David A. Loewenstein

**Affiliations:** ^1^Center for Cognitive Neuroscience and Aging, Department of Psychiatry and Behavioral Sciences, University of Miami Miller School of Medicine, Miami, FL, United States; ^2^Department of Neurology and the Center for Translational Research in Neurodegenerative Disease, University of Florida, Gainesville, FL, United States; ^3^Department of Applied Physiology and Kinesiology, Gainesville, FL, United States; ^4^Department of Neurology and McKnight Brain Institute, University of Florida, Gainesville, FL, United States; ^5^Department of Clinical and Health Psychology, University of Florida, Gainesville, FL, United States; ^6^Department of Psychology, Florida Atlantic University, Boca Raton, FL, United States; ^7^Wien Center for Alzheimer’s Disease and Memory Disorders, Mount Sinai Medical Center, Miami, FL, United States; ^8^Center for Advanced Technology and Education, Florida International University, Miami, FL, United States

**Keywords:** mild cognitive impairment, semantic intrusions, plasma biomarkers, sematic interference, inhibitory control, p-tau amyloid, ApoE, LASSI-L

## Abstract

**Introduction:**

Semantic intrusion errors (SI) have distinguished between those with amnestic Mild Cognitive Impairment (aMCI) who are amyloid positive (A+) versus negative (A−) on positron emission tomography (PET).

**Method:**

This study examines the association between SI and plasma – based biomarkers. One hundred and twenty-eight participants received SiMoA derived measures of plasma pTau-181, ratio of two amyloid-β peptide fragments (Aβ42/Aβ40), Neurofilament Light protein (NfL), Glial Fibrillary Acidic Protein (GFAP), ApoE genotyping, and amyloid PET imaging.

**Results:**

The aMCI A+ (*n* = 42) group had a higher percentage of ApoE ɛ4 carriers, and greater levels of pTau-181 and SI, than Cognitively Unimpaired (CU) A− participants (*n* = 25). CU controls did not differ from aMCI A− (*n* = 61) on plasma biomarkers or ApoE genotype. Logistic regression indicated that ApoE ɛ4 positivity, pTau-181, and SI were independent differentiating predictors (Correct classification = 82.0%; Sensitivity = 71.4%; Specificity = 90.2%) in identifying A+ from A− aMCI cases.

**Discussion:**

A combination of plasma biomarkers, ApoE positivity and SI had high specificity in identifying A+ from A− aMCI cases.

## 1. Introduction

Semantic interference paradigms that measure both proactive semantic interference (PSI) and the failure to recover from proactive semantic interference (frPSI) have been useful to detect biomarker-confirmed preclinical and prodromal Alzheimer’s Disease (AD) and have been related to multiple neuroimaging markers of AD and neurodegeneration among culturally diverse older adults (1–7). Difficulties in correctly recalling information from a second list that is semantically similar to the first set of target words learned is referred to as PSI. FrPSI refers to the inability to recover from initial PSI despite a repeated opportunity to re-learn the List B targets. This is thought to reflect difficulties with inhibition caused by targets in list A as well as potential difficulties with source memory (6, 8, 9).

In contrast, semantic intrusion (SI) errors refer to intrusions of semantically similar list A targets during both the first and second attempts of cued recall of the list B targets. SI on measures of PSI and frPSI likely occur due to deficient self-monitoring and/or reduced semantic inhibitory control and may involve the breakdown of functional subsystems serving medial temporal structures and monitoring systems within the prefrontal lobes (8).

Both frPSI and semantic intrusion errors have been associated with biomarker-confirmed preclinical and prodromal AD using amyloid PET imaging (5, 10). For example, frPSI has shown strong associations with brain amyloid load in otherwise clinically and cognitively unimpaired (CU) community-dwelling older adults (5). Loewenstein and colleagues (10) found that semantic intrusion errors that occurred when a person is challenged by semantic interference (both PSI and frPSI) were substantially more pronounced in persons with amnestic Mild Cognitive Impairment (aMCI) who were Aβ positron emission tomography (PET) positive as opposed to aMCI individuals who were Aβ PET negative. Among asymptomatic middle-aged children of a parent diagnosed with late-onset AD, semantic intrusion errors on frPSI trials were related to deficits in cortico-limbic connectivity (6).

In traditionally used cognitive paradigms (e.g., delayed recall to measure forgetting over a specified time), frequently evidence less sensitivity in detecting early and subtle cognitive breakdowns than previously thought, and limited specificity in preclinical and prodromal AD states (2, 8). To meet the needs of the rapidly evolving AD field, cognitive assessment instruments must demonstrate sufficient scientific rigor including robust sensitivity, specificity, and predictive utility among culturally diverse populations, and importantly, be correlated to definitive AD biomarkers such as β-amyloid and phosphorylated tau (pTau-181) (8).

The pathology of AD is characterized by the abnormal accumulation of amyloid beta protein (Aβ) and phosphorylated tau protein within the neurofibrillary tangles (11, 12). PET scans have made it possible to detect these pathological changes which are considered essential to biologically define AD, per the most recent research framework proposed by the National Institute on Aging and Alzheimer’s Association (NIA-AA) the Amyloid Tau Neurodegeneration [ATN] framework (13, 14). Cerebrospinal fluid (CSF) biomarkers Aβ42, total tau, and p-tau are accepted as the core AD fluid biomarkers recognized by this and other diagnostic frameworks. Other CSF biomarkers have also been strongly related to AD including the Aβ42/Aβ40 ratio, pTau-181, as well as non-specific markers of neurodegeneration including Neurofilament Light protein (NfL) and glial fibrillary acidic protein (GFAP) (15, 16). The relationship between tau markers of AD and tau PET imaging has been amply described by Coomas and colleagues, whose findings indicate that both plasma pTau181 and tau PET are effective in identifying Aβ pathology, but tau PET is better at monitoring disease stage and clinical progression (17).

While CSF levels of Aβ and tau have long been recognized as the most valid fluid markers of neuropathology, this can be difficult to obtain (18). More recently, highly sensitive methods have enabled the successful detection of protein fragments in the peripheral plasma. Thus, using advanced microarray plasma assays, the Aβ42/Aβ40 ratio (19–21), and pTau-181 (22–26) are now reliably detected in the plasma. This advancement is largely due to the introduction of SiMoA technology that employs ultra-sensitive assays to detect protein molecules in the blood (27).

In the current study, we examined the cognitive performance of Amyloid negative (A−) CU controls in comparison to Amyloid positive (A+) aMCI (prodromal AD) individuals, as well as persons with A− aMCI (cognitive impairment, not AD). This represents a significant expansion of our prior studies by determining whether plasma markers of AD and neurodegeneration were associated with cognitive performance in each diagnostic group. We also explored whether a combination of blood-based biomarkers and performance on the LASSI-L could differentiate between A+ and A− aMCI participants. Our hypotheses were that (1) that LASSI-L SI errors would differentiate between A+ aMCI and A− aMCI participants, (2) that pTau181 would be most predictive of SI in LASSI-L PSI and frPSI trials, and (3) that amyloid PET positivity among aMCI participants would best be predicted by biomarkers such pTau181 and LASSI-L SI errors.

## 2. Methods

### 2.1. Sample

We recruited 128 adults aged 60 and above, who underwent an extensive clinical evaluation and standardized neuropsychological testing as part of the 1Florida Alzheimer’s Disease Research Center (ADRC) protocol. All participants also underwent amyloid PET imaging that was visually rated by an experienced neuroradiologist as A+ or A−. A high degree of reliability has been shown for these visual amyloid PET ratings (28). Each participant had blood drawn for biomarker analysis using SiMoA for plasma pTau-181, Aβ42/Aβ40ratio, NfL, and GFAP.

### 2.2. Amnestic MCI group

One hundred three individuals had a clinical diagnosis of aMCI, as established by using the following criteria: (a) a memory complaint preferably confirmed by a knowledgeable informant; (b) a Global Clinical Dementia Rating Scale (CDR) (29) Score of 0.5; (c) did not meet criteria for a Major Cognitive Disorder based on DSM-5 criteria (30); (d) scored 23 or higher on the Mini-Mental State Exam (MMSE) (31) and; (e) scored at least one standard deviation (SD) below average on either the Hopkins Verbal Learning Test-Revised (HVLT-R) (32) immediate recall, HVLT-R delayed recall or delayed recall on the NACC (33) story passages using extensive local normative data based on age, education, sex, and language of testing (English versus Spanish). Other non-memory measures [such as the Trail Making Test-B (34) and Category Fluency (35)] could demonstrate one SD or greater above or below the mean, but a memory deficit had to be established in order to be categorized as aMCI. Of the 103 participants with aMCI, 42 were A+ on PET scan and 61 were A−.

### 2.3. Cognitively unimpaired group

We included 25 CU controls who (a) had no subjective memory concerns corroborated by a knowledgeable informant; (b) had a Global CDR score of 0; (c) did not meet criteria for either Major or Minor Neurocognitive Disorder by DSM-5 criteria; (d) had scores on the HVLT-R immediate and delayed memory, NACC delayed recall, Trail Making Test A and B, Category Fluency and the Stroop Color Word test all within normal limits. The normal scores were less than 1SD below expected levels for age, education, sex, and language of testing (English versus Spanish). It is important to note that all CU individuals had A-scans. Plasma samples were analyzed using the same procedures as in the aMCI group.

### 2.4. Loewenstein-Acevedo scales for semantic interference and learning

The LASSI-L was not used for diagnostic determination in this study to avoid potential issues of criteria contamination and circularity. The LASSI-L employs controlled learning and cued recall in an effort to maximize the storage of a list of to-be-remembered target words belonging to three distinct semantic categories (fruits, clothing, and musical instruments) (2, 5). Participants were tested in their preferred language (English or Spanish) by trained bilingual psychometrists. The LASSI-L has been shown previously to be culturally fair and valid in either language (7, 36). Moreover, since the language of administration does not impact scores, English and Spanish administrations can be pooled for data analysis (7). During the administration of the LASSI-L, the examinee is instructed to remember a list of 15 common words (List A) representing three semantically distinct categories (i.e., fruits, musical instruments, and articles of clothing). The words are presented one at a time on cards and are read out loud by the participants. Once all 15 words from the first list (List A) have been presented, there is a free recall trial, followed by cued recall trials for each of the three categories. List A is presented again, and an additional cued recall trial for each category is conducted.

A unique aspect of the LASSI-L paradigm is the presentation of a second competing list of to-be-remembered words as a way to elicit a considerable amount of PSI. A second list of different words (List B) from the same semantic categories is presented immediately after the second trial of List A. This is followed by a free recall trial and cued recall trials (one per category-Cued B1). Lastly, List B is presented for a second time, with another round of cued recall trials (Cued B2) as outlined in Crocco et al. (37). Unlike other traditional memory assessment paradigms, the re-administration and subsequent recall of this second list of words measure the individual’s ability to recover from the effects of PSI (frPSI) (8). An individual can have PSI but with another administration of List B, can recover from PSI because of the additional learning trial. Thus, there are cases with initial PSI that recover from PSI but lack the frPSI effect. While there is an additional retroactive semantic interference condition, this was not a focus of the current study since this does not appear to have a significant discriminatory ability (8, 38).

In the present investigation, we examined LASSI-L measures such as correct responses on Cued B1 and Cued B2 subject to PSI and frPSI. We were particularly interested in examining semantic intrusion errors on the LASSI-L Cued B1 and Cued B2, since these measures have been previously shown to be particularly sensitive to amyloid load (8, 38). These intrusion errors primarily involve words from List A as both lists share semantically similar target items and other semantically related words that were not on List A (9). Intrusion errors produced on the Cued B1 and Cued B2 trials are extremely sensitive to PSI and frPSI deficits thought to reflect deficits in self-monitoring and semantic inhibitory control (2, 9, 39). These errors are measured by counting the total raw number of non-target words that were recalled on each cued recall trial of List B.

### 2.5. Analysis of amyloid pet imaging scans

Using a methodology similar to that described by Seibly and colleagues (40), tracer uptake was assessed in six cortical regions (orbitofrontal, frontal, parietal, lateral temporal, occipital, and precuneus/posterior cingulate cortex), combining values from the left and right hemispheres by two experienced raters who were blind to the cognitive and clinical diagnoses. 18F-florbetaben (FBB) PET/CT scans were qualitatively analyzed and classified based on the brain amyloid plaque load (BAPL) scoring scheme. BAPL 1 scans were considered negative for amyloid brain deposition (A−) and BAPL 2 or BAPL 3 scans were considered as positive for amyloid brain deposition (A+). A final dichotomous (A+ versus A−) diagnosis was rendered. Using this methodology, Loewenstein and colleagues (10) reported high interrater reliability for amyloid visual reads. Visual amyloid reads are considered the gold standard in the field (28).

### 2.6. Analysis of blood-based biomarkers using SiMoA digital immunoassays

Baseline blood samples were collected in K2EDTA lavender-top blood collection tubes (Fisher catalog #265732) and were centrifuged at room temperature within 1 h of blood draw to separate the plasma. Plasma was aliquoted into polypropylene cryovials and stored at −80°C. To ensure tracking of freeze-thaw cycles of plasma aliquots, a single plasma aliquot (approximately 500 microliters) was thawed, centrifuged at 10,000 rcf for 5 min at 4°C, and sub-aliquoted into single-use aliquots for Quanterix SiMoA assays with storage at −80°C until used. Samples were analyzed in duplicate using a Quanterix SR-X Analyzer with SiMoA Assay kits to measure concentrations of plasma pTau-181 (pTau-181 Advantage V2 Kit, Item 103,714), Aβ42 and Aβ40 (Neurology 3-Plex A Advantage Kit, Item 101,995), and NfL and GFAP (Neurology 3-Plex B Advantage Kit, Item 103,520). Plasma samples were randomized and run while blinded to diagnostic status or any other study variables. The manufacturer’s recommendations were followed for each assay run. Concentrations of proteins in the plasma (pg/mL) are reported as an average of the duplicates. All samples employed in this investigation were required to have a coefficient of variation of less than 20% and assay kit controls were within the manufacturer’s acceptable range ApoE genotyping for our ADRC sample was conducted at the National Alzheimer’s Coordinating Center (NACC) through the National Centralized Repository for Alzheimer’s Disease and Related Dementias (NCRAD). If one or more alleles were ɛ4 positive, the variable was coded as “1.” Negative results were coded as “0,” which creates an ideal analysis for linear and logistic regression models described below.

### 2.7. Statistical analyses

All participants had the amyloid PET imaging, LASSI-L variables, and the full complement of valid p-tau-181, AB40, AB42, NfL GFAP, and ApoE genotype. SPSS Version 28 was the statistical software package employed in the current investigation. Since there were three diagnostic groups with interval-level data, a series of wone-way analyses of variance (ANOVA) were performed. Following a statistically significant F at *p* < 0.05, post-hoc test of means was conducted using the Tukey Honestly Significant Difference Test (HSD) with statistical significance set at *p* ≤ 0.05. Categorical variables were examined using Chi-square analyses with the criterion for statistical significance also set at *p* < 0.05. Since the two aMCI groups (A+ and A−) had statistically different mean MMSE scores, subsequent covariate analyses adjusting for MMSE were conducted on blood-based biomarkers and LASSI-L scores and there were no differences in the obtained results.

Since only SI errors on the LASSI-L statistically differentiated between aMCI groups, we employed stepwise regression to determine those plasma biomarkers, ApoE ɛ4 status, as well as demographic factors that predicted semantic intrusion errors among aMCI participants on PSI and frPSI measures of the LASSI-L. Further stepwise logistic regression determined the combination of blood-based biomarkers, LASSI-L, ApoE ɛ4 status, and demographic factors that best differentiated between aMCI A+ and A− groups. It should be noted that forward and backward entry yielded identical results. Additionally, the results remained the same when demographic factors were entered first as a block before the other variables.

## 3. Results

There were no statistically significant group differences between Cognitively Unimpaired (CU A−, aMCI A−) or aMCI A+ groups with regards to age, years of education, or Hispanic/Latino ethnicity (Table 1). As expected, there were group differences in frequency of the ApoE ɛ4 allele [*X*2 (df = 2) = 33.35; *p* < 0.001] and MMSE scores [*F* (2,125 = 23.28, *p* < 0.001)]. There were also statistically significant group differences in pTau-181 [*F* (2,125) = 22.63; *p* < 0.001], Aβ42/40 ratio [*F* (2,125) = 6.22; *p* = 0.003]; and GFAP [*F*(2,125) = 9.03; *p* < 0.001], but not NfL [*F*(2,125) = 2.32; *p* = 0.105]. There were also statistically significant group differences in LASSI-L performance on Cued B1 total correct recall [*F*(2,125) = 18.30; *p* < 0.001], Cued B1 Intrusions, [*F*(2,125) = 12.76; *p* < 0.001] Cued B2 total correct recall [*F*(2,125) = 13.71; *p* < 0.001], and Cued B2 Intrusions [*F*(2,125) = 12.31; *p* < 0.001]. Post-hoc tests of means indicated that CU individuals had the highest MMSE scores, followed by the aMCI A− group. The lowest MMSE scores were obtained by the aMCI A+ group. The A+ aMCI group had higher concentrations of pTau181 and GFAP relative to aMCI A− and CU A− groups; the latter two had statistically equivalent scores. Consistent with prior findings, A+ aMCI participants also evidenced a lower Aβ42/40 ratio than CU A− participants. Individuals who were A+ aMCI differed from their A− aMCI counterparts with regards to semantic intrusions, but not total correct responses on subtests tapping PSI and frPSI.

**Table 1:** Descriptive characteristics of the sample.

	CU A− (*n* = 25)	aMCI A− (*n* = 61)	aMCI A+ (*n* = 42)	*F*-value	*p* or *X*^2^ value	Value of *p* for A+ versus A−comparisons adjusting for MMSE	Value of *p*
Age (range 57–98)	70.94 (SD = 6.0)	73.47 (SD = 7.4)	73.31 (SD = 7.5)	1.193	0.397	NA	
Education (range 6–22)	15.92 (SD = 3.1)	15.03 (SD = 3.6)	14.79 (SD = 3.2)	0.941	0.303	NA	
Sex % Females	76.0%	50.8%	61.9%	4.83	0.089	NA	
% Hispanic	60.0%	54.1%	69.0%	2.23	0.314	NA	
ApoE ɛ4 positivity	16.0%	18.0%	69.0%	33.35	<0.001	NA	
MMSE (range = 23–30)	29.36^a^ (SD = 0.85)	27.98^b^ (SD = 1.6)	26.45^c^ (SD = 2.1)	23.38	<0.001	NA	
Plasma pTau181 pg./ml range (0.714–6.752)	1.787^a^ (SD = 0.901)	2.186^a^ (SD = 1.140)	3.507^b^ (SD = 1.335)	22.37	<0.001	**19.403**	**p < 0.001**
Plasma Aβ42/Aβ40pg/ml range (0.019–0.075)	.045^a^ (SD = 0.006)	.043^ab^ (SD = 0.009)	.039^b^ (SD = 0.007)	6.22	0.003	3.858	*p* = 0.053
Plasma GFAP pg./ml range (38.801–807.92)	157.132^a^ (SD = 71.615)	171.771^a^ (SD = 110.680)	254.934^b^ (SD = 127.084)	8.029	<0.001	**6.917**	***p* = 0.01**
Plasma NFL pg./mlrange (4.001–42.298)	12.100 (SD = 5.270)	15.164 (SD = 9.530)	16.395 (SD = 9.530)	2.315	0.103	0.072	*p* = 0.789
LASSI-L CUED B1 (PSI) range (0–12)	8.79^a^ (SD = 2.29)	5.41^b^ (SD-2.74)	5.52^b^ (SD = 2.17)	18.30	<0.001	0.347	*p* = 0.506
LASSI-L CUED B2 (frPSI) range (3–15)	11.56^a^ (SD = 2.04)	8.66^b^ (SD = 2.9)	8.50^b^ (SD = 2.19)	13.76	<0.001	0.576	*p* = 0.409
LASSI Cued B1 Intrusions range (0–12)	2.60^a^ (SD = 2.43)	3.56^a^ (SD = 2.91)	5.90^b^ (SD = 3.06)	12.78	<0.001	**6.669**	***p* = 0.011**
LASSI Cued B2 Intrusions range (0–11)	1.72^a^ (SD = 1.70)	2.67^a^ (SD = 2.59)	4.52^b^ (2.52)	12.31	<0.001	**5.170**	***p* = 0.025**

Means with different superscripts are statistically significant at *p* < 0.05 by the Tukey’s HSD test. Bold values are statistically significant results after adjusting for covariates in the model.

Since participants differed on their global MMSE scores, it might be argued that these group differences may have impacted LASSI-L performance. However, even after adjusting for differences in global mental status as measured by MMSE scores, using ANOVA only semantic intrusion errors on Cued B1 and Cued B2 indices remained statistically significant between groups and differentiated A+ aMCI from A− aMCI participants. In addition, adjusting for ApoE e4 status did not change the obtained findings.

Since only LASSI-L Cued B1 and Cued B2 semantic intrusions differentiated between A+ and A− aMCI groups, we determined those demographics, factors, ApoE4 and plasma biomarkers that could predict these types of these errors. Among aMCI participants stepwise linear regression revealed that only p-tau 181 and ApoE status were predictive of Cued B1 semantic intrusion Total *R* = 0.357 [*F* (2,100) = 7.30; *p* < 0.001] (Table 2). For Cued B2 intrusions, plasma pTau-181 and positive ApoE4 genotype were also the only variables that entered into the model, with total *R* = 0.376 [*F* (1,100) = 8.25; *p* < 0.001] (Table 3). Specific beta and standardized beta weights are presented in Tables 2, 3. No demographic variables such as age education, sex, or Hispanic/Latino ethnicity entered into the model.

**Table 2:** Plasma biomarkers as predictors of LASSI-L Cued B1 intrusions-stepwise linear regression.

Coefficients								
	Model	Unstandardized coefficients		Standardized coefficients	*t*	Sig.	95.0% confidence interval for B	
		B	Std. Error	Beta			Lower bound	Upper bound
1	(Constant)	2.616	0.665		3.933	<0.001	1.297	3.936
	pTau 181	0.697	0.218	0.303	3.196	0.002	0.264	1.129
2	(Constant)	2.415	0.663		3.644	<0.001	1.100	3.73
	pTau181	0.591	0.221	0.257	2.675	0.009	0.153	1.03
	ApoE ɛ4 positivity	1.258	0.623	0.194	2.019	0.046	0.022	2.494

**Table 3:** Plasma biomarkers as predictors of LASSI-L Cued B2 semantic intrusion errors-stepwise linear regression.

Coefficientsᵃ								
	Model	Unstandardized coefficients		Standardized coefficients	*t*	Sig.	95.0% confidence interval for B	
		B	Std. Error	Beta			Lower bound	Upper bound
1	(Constant)	1.770	0.566		3.128	0.002	0.648	2.893
	pTau181	0.608	0.185	0.310	3.280	0.001	0.240	0.976
2	(Constant)	1.577	0.561		2.812	0.006	0.464	2.689
	pTau181	0.506	0.187	0.258	2.710	0.008	0.136	0.877
	ApoE ɛ4 positivity	1.212	0.527	0.219	2.300	0.024	0.167	2.257

Finally, an attempt was made to predict amyloid status among aMCI participants by examining demographic variables, blood-based biomarkers, ApoE4 status and LASSI-L intrusion scores. Using stepwise logistic regression, independent predictors of ApoE4 positivity, pTau-181 and LASSI-Cued B1 intrusions resulted in Sensitivity = 71.4%; Specificity = 90.2% and Overall Classification = 82.5% in distinguishing between aMCI participants who were A+ and A−. All demographic variables (age, sex, years of education, Hispanic ethnicity, MMSE) and other variables (NfL, GFAP) were non-significant when entered into the model (See Tables 4, 5).

**Table 4:** Multivariate prediction of amyloid positive and amyloid negative PET among aMCI using a step-wise logistic regression (Sensitivity = 71.4%; Specificity = 90.2% Overall Classification = 82.5%).

Variables in the equation							
		B	S.E	Wald	df	Sig.	Exp(B)
Step 1ᵃ	ApoE ɛ4 positivity (1)	−2.316	0.472	24.137	1	<0.001	0.990
	Constant	1.347	0.311	18.722	1	<0.001	3.846
Step 2ᵇ	pTau181	−0.842	0.229	13.565	1	<0.001	0.431
	ApoE ɛ4 positivity (1)	−2.315	0.534	18.805	1	<0.001	0.099
	Constant	3.633	0.734	24.510	1	<0.001	37.824
Step 3ᶜ	LASSI-L Cued B1 Intrusions	−0.191	0.092	4.322	1	0.038	0.826
	pTau181	−0.782	0.230	11.612	1	<0.001	0.457
	ApoE ɛ4 positivity (1)	−2.272	0.554	16.793	1	<0.001	0.103
	Constant	4.358	0.881	22.440	1	<0.001	78.064

All demographic variables (age, sex, years of education, Hispanic ethnicity, MMSE) and other variables (NfL, GFAP) were non-significant when entered into the model.

**Table 5:** Statistically significant predictors of amyloid positive status among participants with aMCI.

Classification table				
	Observed	Correct classification		Percentage correct
		A+ (*n* = 42)	A− (*n* = 61)	
Step 1	ApoE positivity	29/42		69.0%
			50/61	82.0%
	**Overall Percentage**			**76.7%**
Step 2	pTau181	29/42		69.0%
	ApoE ɛ4 positivity		53/61	86.9%
	**Overall Percentage**			**79.6%**
Step 3	LASSI-L Cued B1 Intrusions	30/42		71.4%
	pTau181 ApoE ɛ4 positivity		55/61	90.2%
	**Overall Percentage**			**82.5%**

All demographic variables (age, sex, years of education, Hispanic ethnicity, MMSE) and other variables (NfL, GFAP) were non-significant when entered into the model.

## 4. Discussion

Previous findings have indicated that LASSI-L semantic intrusion (SI) errors have distinguished between amnestic aMCI persons who are A+ from aMCI participants that are A− (5, 10). Similar results have been obtained when A− groups have been divided into SNAP (suspected Non-AD Pathology), other neurological disorders, or neuropsychiatric disorders (41). The present study provided a unique and unprecedented opportunity to study how SI errors on the LASSI-L, reflective of self-monitoring deficits and lack of inhibitory control (8–10, 39) related to plasma biomarkers of AD as well as non-specific markers of neurodegeneration, and genetic risk for AD.

As with previous research, it was only SI errors (rather than correct responses) on trials susceptible to proactive semantic interference (PSI) and the failure to recover from proactive semantic interference (frPSI) that were associated with amyloid positivity on PETpT181. Interestingly, t A− aMCI and A− CU participants did not differ with regards to these types of errors. This is consistent with the notion that SI errors likely represent unique cognitive marker of incipient AD that represent breakdowns in self-monitoring, and semantic inhibitory control (2, 8, 38).

The finding that SI errors were related to pTau181 and genetic risk for AD as determined by the presence of ApoE ɛ4, in a manner that was significantly more prevalent in A+ versus A− aMCI participants further suggests that SI is a cognitive marker of AD pathology that can be measured during the pre-dementia state. The finding that pTtau181 was the most sensitive marker of AD pathology is consistent with previous reports that plasma pTau-181, p-Tau-217, and p-Tau-231 may all be sensitive indicators of underlying AD pathology (22–26). While not specifically related to deficits in SI errors on the LASSI-L, GFAP clearly differentiated A+ aMCI from A− aMCI participants and CU. Consistent with the data depicted in Table 1, plasma GFAP has increasingly been shown to be related to amyloid PET and CSF markers of AD pathology and progression to AD dementia (15, 19).

Despite bordering on statistical significance, the finding that Aβ42/Aβ40 could distinguish between A+ aMCI participants and CU controls is also consistent with previous findings (19, 21) although A+ and A− aMCI groups could not be distinguished. This may have occurred given increasing evidence that Aβ42 and Aβ40 may not be as accurately detected in the plasma with SiMoA relative to other techniques such as mass spectroscopy (27). Bilgel and colleagues examined the longitudinal changes in plasma biomarkers related to AD neuropathology and neurodegeneration compared to amyloid plaques. The findings suggest that plasma biomarkers, particularly pTau231 and GFAP, closely align with changes in brain amyloid levels over time. Plasma Aβ42/Aβ40 may decline prior to the emergence of brain amyloid plaques, while other plasma biomarkers show more pronounced changes closer to the accumulation of brain amyloid (42). This may also account for the finding that the AB42/AB42 ratio did not predict SI in regression equations. Clearly, this is an area that is worthy of further research. It was somewhat unexpected that NfL did not reach statistical significance in the three-group comparisons. However, post-hoc analyses did show statistically significant group differences when two group comparisons between the most extreme groups, A+ aMCI and A− CU individuals, were examined. We and others have previously shown that NfL levels are associated with AD as well as other neurological disease states (43, 44). It is important to note that despite the inclusion of demographic variables and all blood-based biomarker predictor variables in step-wise regression equations, only pTau181 was predictive of outcome (see Tables 4, 5).

A particularly interesting finding of the current investigation is that a combination of pTau181, ApoE ɛ4 positivity, and LASSI-Cued B1 intrusion errors predicted amyloid status in individuals with aMCI, correctly identifying over 90% of A− cases and over 71% of A+ cases. The fact that these particular blood-based biomarkers in conjunction with a measure of semantic inhibitory control demonstrated such high levels of specificity indicates that multivariate approach as such can be used to quickly identify those that might benefit from more extensive work-up or as a method of screening individuals for emerging clinical trials. Strengths of the current study include a carefully evaluated sample of older adults with the full complement of SiMoA plasma-based biomarkers and ApoE ɛ4 genotype that could be compared to amyloid PET data. Moreover, there were no statistically significant group differences on important demographic variables such as age, education, sex, and Hispanic/Latino ethnicity. Another strength was the examination of the associations between a combination of plasma-based biomarkers, ApoE ɛ4, and SI errors, which evidenced excellent overall classification based on multivariate prediction.

Limitations of the study include a relatively modest number of cognitively unimpaired persons, and that these were predominantly female. Further, the cross-sectional nature of the investigation did not allow for the prediction of plasma biomarkers as they related to cognitive decline. Finally, we were unable to obtain CSF on this ADRC sample that could be compared to plasma-based biomarker results. Future studies with larger sample sizes and longitudinal follow-up will shed greater light on the predictive properties of both plasma biomarkers and deficits in self-monitoring and semantic inhibitory control (reflected by SI intrusions) as they relate to rates of clinical progression over time. These multivariate predictive models using novel cognitive challenge tests, genetic, and other blood-based biomarkers which are more convenient and easier to obtain, offer promise for future clinical research.

## Data availability statement

The raw data supporting the conclusions of this article will be made available by the authors, without undue reservation.

## Ethics statement

This study is an IRB-approved investigation at the University of Miami Miller School of Medicine and Mt. Sinai Medical Center. All procedures performed met all national and international standards for the protection of human subjects. Informed consent was collected from all participants, who were compensated for their participation.

## Author contributions

RC and DL developed the study concept, design, and completed the data analysis. AO took the lead in preparing the manuscript for final submission. RC, AO, EC, DH, KM, RD, DV, SD, GS, ES, MR, WB, MA, YB, YF, and DL provided critical feedback and helped to shape the research, analysis, and manuscript. All authors contributed to the article and approved the submitted version.

## Funding

This research was funded by the National Institute of Aging Grant 1 R01 AG047649-01A1 (RC, PI), 1 R01 AG047649-01A1 (DL, PI); as well as by the 1Florida Alzheimer’s Disease Research Center 5 P50 AG047726602 and 1P30AG066506-01 1 8AZ (GS, Interim PI). The sponsors had no role in the design and conduct of the study; in the collection analysis, and interpretation of data; in the preparation of the manuscript; or in the review or approval of the manuscript.

## Conflict of interest

The LASSI-L measure was developed by DL and RC at the University of Miami, who holds intellectual property rights.

The remaining authors declare that the research was conducted in the absence of any commercial or financial relationships that could be construed as a potential conflict of interest.

## Publisher’s note

All claims expressed in this article are solely those of the authors and do not necessarily represent those of their affiliated organizations, or those of the publisher, the editors and the reviewers. Any product that may be evaluated in this article, or claim that may be made by its manufacturer, is not guaranteed or endorsed by the publisher.
